# A new species of *Diartiger* Sharp (Staphylinidae, Pselaphinae, Clavigeritae) from the Fengyangshan – Baishanzu Nature Reserve, East China

**DOI:** 10.3897/zookeys.419.7867

**Published:** 2014-06-24

**Authors:** Zi-Wei Yin, Li-Zhen Li

**Affiliations:** 1 Department of Biology, College of Life and Environmental Sciences, Shanghai Normal University, 100 Guilin Road, Shanghai, 200234, P. R. China

**Keywords:** Taxonomy, Clavigeritae, *Diartiger*, new species, myrmecophily, *Lasius*, Asia

## Abstract

A new clavigerine pselaphine, *Diartiger zhejiangensis* Yin & Li, **sp. n.**, from the Fengyangshan – Baishanzu Nature Reserve, southern Zhejiang, is described, illustrated, and compared with congeners. The species is hosted by ants in the genus *Lasius*. The key to *Diartiger* species from China is modified to accommodate the new species.

## Introduction

In a recent publication (Yin and Li 2013), the relationship between *Diartiger* Sharp and *Microdiartiger* Sawada was discussed, the synonymy of the two genera defended, and two new species were described from Anhui, East China. As a result of that work, the Chinese *Diartiger* includes four species, with *Diartiger dentatus* Yin & Li, *Diartiger kunmingensis* Nomura, and *Diartiger yaoluopingensis* Yin & Li placed in the *Diartiger fossulatus* group, and *Diartiger songxiaobini* (Yin & Li) in the *Diartiger japonicus* group.

During a survey (26.iv–03.v.2014) of the staphylinid beetles at the Fengyangshan – Baishanzu Nature Reserve, Zhejiang, three adults of an undetermined *Diartiger* were discovered, two in *Lasius* colonies, and one from a sifted litter sample. This species can be separated from all known congeners by a unique combination of external characters, and is formally described herein. The key to *Diartiger* species occurring in China is modified to accommodate the new species.

## Materials and methods

All material treated in the present study is housed in the Insect Collection of Shanghai Normal University, Shanghai, China (SNUC).

This study is based on three adults collected during a recent survey of the staphylinid beetles at the Fengyangshan – Baishanzu Nature Reserve by our lab students Zhong Peng and Xiao-Bin Song. The two *Diartiger* adults and host ants from Baishanzu Nature Reserve were transported to our lab alive for behavioral observations and photos.

Observations and dissections were carried out using an Olympus SZ61 Stereo microscope. Dissected parts were preserved in Euparal mounting medium (BioQuip Products, Inc., CA, U.S.A.) on plastic slides that were placed on the same pin with the specimen. Digital habitus images of dead and live adults were created using a Canon 7D digital camera in conjunction with a Canon MP-E 65mm f/2.8 1-5X Macro Lens, a Canon MT-24EX Macro Twin Lite Flash, and a flash light diffuser made by parchment paper. Images of the dissected parts were made using a Canon G9 camera mounted by hand on a Olympus CX31 microscope. Zerene Stacker version 1.04 was used for image stacking, and all images were edited and grouped in Adobe Photoshop CS5 Extended (version 12.0).

The following abbreviations are applied: AL–length of the abdomen along the midline; AW–maximum width of the abdomen; EL–length of the elytra along the sutural line; EW–maximum width of the elytra; HL–length of the head from the anterior clypeal margin to the occipital constriction; HW–width of the head across eyes; PL–length of the pronotum along the midline; PW–maximum width of the pronotum. Length of the body is a combination of HL + PL + EL + AL.

## Taxonomy

### Diartiger zhejiangensis

Taxon classificationAnimaliaColeopteraStaphylinidae

Yin & Li sp. n.

http://zoobank.org/20113B64-96ED-4879-AB1D-D23243DBEF12

Figs 1 – 3

#### Type material

(2 ♂♂, 1 ♀)**. Holotype: CHINA:** ♂, labeled ‘China: S. Zhejiang, Qingyuan, Baishanzu N.R., 27°45'14"N, 119°11'55"E, Fagaceae forest, ant nest in rotten wood, 1650 m, 01.v.2014, X.-B. Song leg. / HOLOTYPE [red], *Diartiger zhejiangensis* sp. n., det. Yin & Li, 2014, SNUC’. **Paratypes: CHINA:** 1 ♀, same area and date, except ‘27°45'27"N, 119°12'05"E, mixed leaf litter, sifted, 1700 m, Z. Peng leg.’; 1 ♂ [disarticulated specimen preserved in Euparal on plastic slides], labeled ‘China: S. Zhejiang, Longquan, Fengyang Shan, creek valley nr. hotel, 27°54'42"N, 119°11'52"E, *Rhododendron* and fir forest, ant nest in erect rotten fir trunk, 1250 m, 28.iv.2014, Peng leg.’. Each paratype bears a type label similar to that of the holotype except ‘PARATYPE [yellow]’.

#### Description.

**Figure 1.**
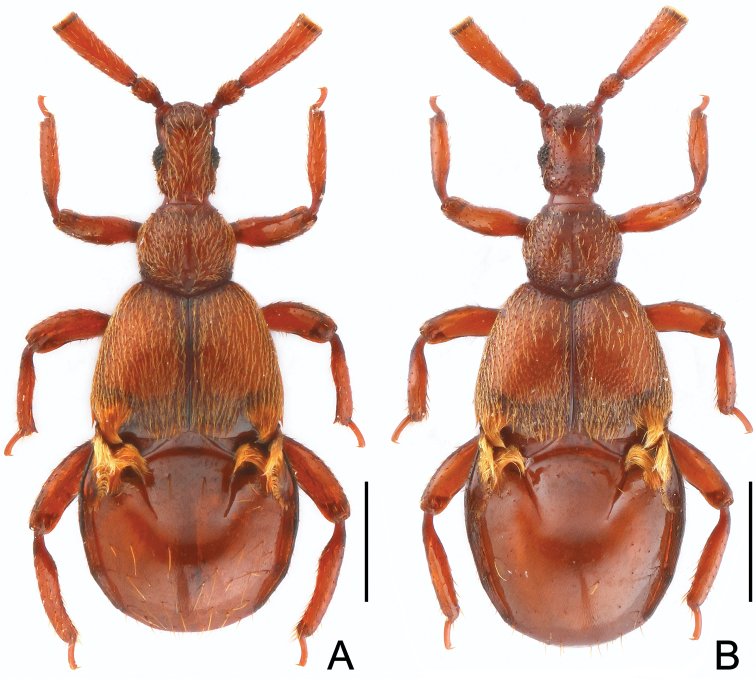
Habitus of *Diartiger zhejiangensis*. **A** Male **B** Female. Scales: 0.5 mm.

**Figure 2.**
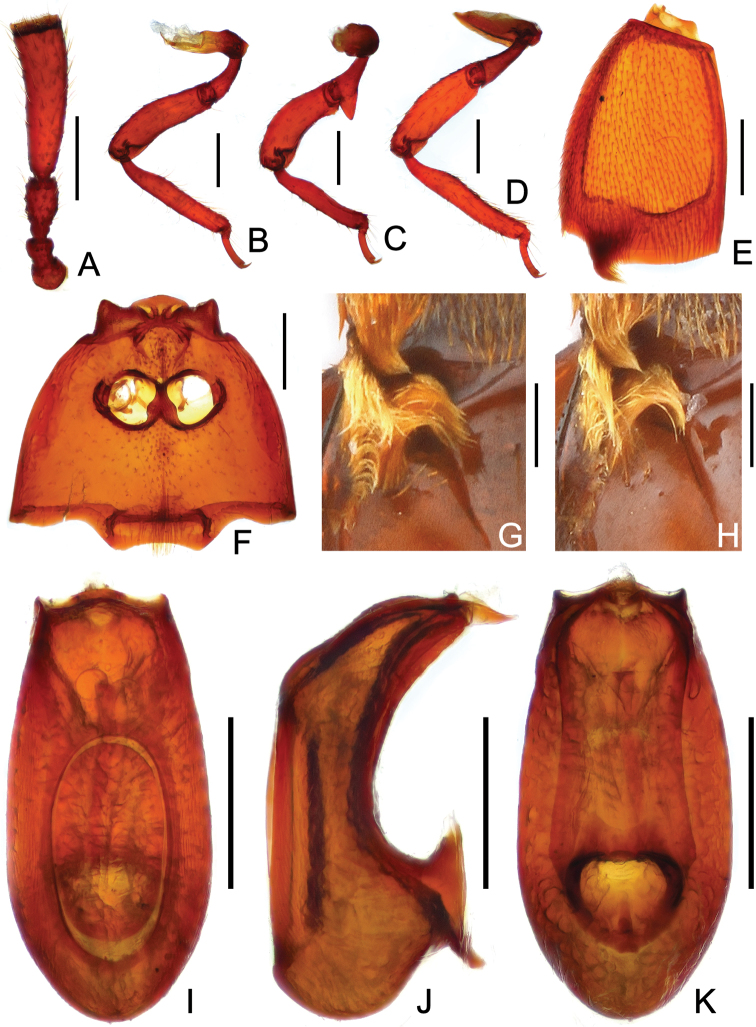
Diagnostic features of *Diartiger zhejiangensis* (**A–G, I–K** male **H** female). **A** Antenna **B** Fore leg **C** Mid leg **D** Hind leg **E** Left elytron **F** Meso- and metaventrites **G–H** Trichomes of elytra and composite tergite **I** Aedeagus, in dorsal view **J** Same, in lateral view **K** Same, in ventral view. Scales: 0.2 mm.

Male. Body (Fig. 1A) length 2.14–2.15 mm; reddish brown. Head longer than wide, HL 0.39–0.40 mm, HW 0.27–0.28 mm; clypeus with slightly angularly rounded anterior margin; eyes each composed of about 20 facets; antennomeres IV (Fig. 2A) more than twice length of III, with truncate apex. Pronotum about as long as wide, PL 0.37–0.38 mm, PW 0.39–0.41 mm. Elytra (Fig. 2E) wider than long, EL 0.53–0.56 mm, EW 0.80–0.82 mm; lacking microsculpture; with small tuft of setae at posterolateral margins, and bigger triangular, posteriorly-narrowed trichomes at posterior margins. Metathoracic wings fully-developed. Mesoventrite (Fig. 2F) lacking median carina, metaventrite (Fig. 2F) slightly convex, both meso- and metaventrites with row of setae along midline. Profemora (Fig. 2B) with long ventral setae at base, protibiae narrowed at base and thickened from basal third toward apex; mesotrochanters (Fig. 2C) with large, bluntly triangular ventral spine, mesofemora lacking spine; mesotibiae with small apical spur; metatibiae (Fig. 2D) lacking spine or modification. Abdomen slightly wider than long, AL 0.82–0.84 mm, AW 0.88–0.92 mm; composite tergite with transverse basolateral trichomes curved mesally, first pair of paratergites with linear trichomes (Fig. 2G). Aedeagal length 0.50–0.52 mm; median lobe (Fig. 2I–K) with sinuate apical margin in dorso-ventral view.

Female. General form (Fig. 1B) and trichomes on elytra and composite tergite (Fig. 2H) similar to those of male; fourth antennomeres slightly shorter, mesotrochanters and mesotibiae lacking spine; abdomen relatively larger. Each eye composed of about 20 facets. Measurements: BL 2.23 mm, HL 0.41 mm, HW 0.28 mm, PL 0.39 mm, PW 0.43 mm, EL 0.55 mm, EW 0.83 mm, AL 0.88 mm, AW 0.92 mm.

#### Comparative notes.

This species is placed as a member of the *Diartiger fossulatus* group. Males are most similar to those of *Diartiger dentatus* in possessing relatively short antennomeres III, and in similarities of trichomes on the posterior margins of the elytra and the base of the composite tergite, and in the aedeagal form. The two species can be readily separated by the lack of spines on the mesofemora and middle of the mesotibiae, and lack of a small tubercle on the ventral margin of the metacoxa in *Diartiger zhejiangensis*, while *Diartiger dentatus* possesses a sharp basal spine on the ventral margin of the mesofemur, the mesotibia possesses a triangular spine at the middle, and the metacoxa possesses a small tubercle on the ventral margin.

#### Distribution.

East China: Zhejiang.

#### Bionomics.

**Figure 3.**
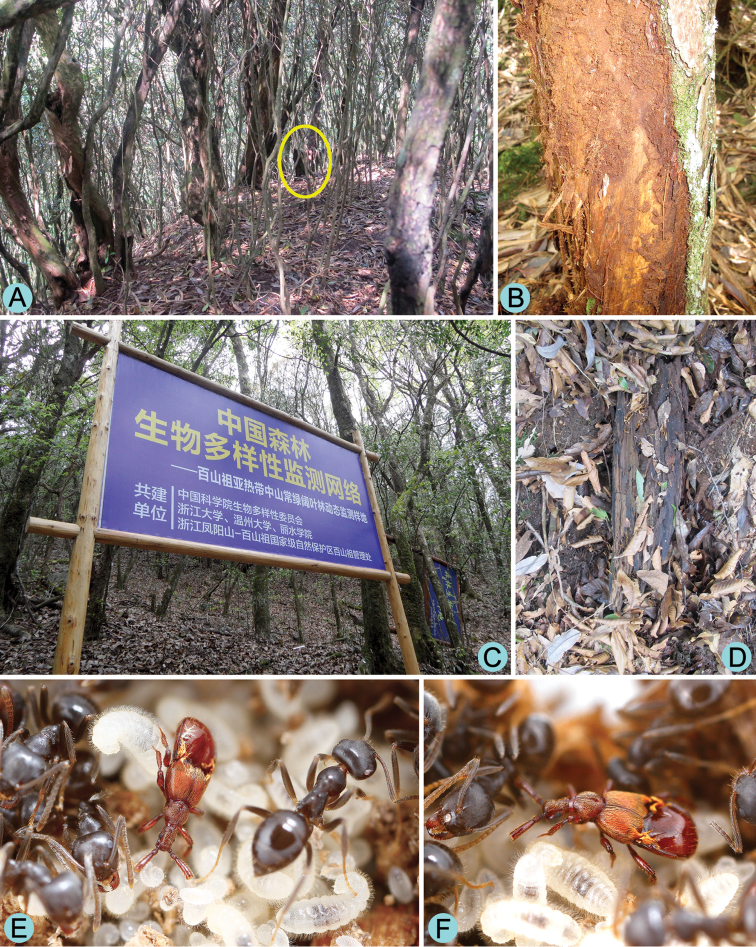
Habitat and host of *Diartiger zhejiangensis*. **A** General habitat of the *Rhododendron* and fir forest at Fengyang Shan Nature Reserve **B** Rotten trunk where the female paratype was collected **C** General habitat of the Fagaceae forest at Baishanzu Nature Reserve **D** Rotten wood where the holotype was collected **E–F** Living male adult of *Diartiger zhejiangensis* with host ants.

The male holotype was collected from a colony of *Lasius* cf. *koreanus* (det. Maruyama, pers. comm. 2014) nesting in rotten wood in a predominantly Fagaceae forest (Fig. 3C, D). The female paratype from Baishanzu was collected from a sifted litter sample. The other female paratype collected from Fengyang Shan was found in a small colony of the same *Lasius* species nesting under bark of a standing rotten fir trunk in a *Rhododendron* and fir forest (Fig. 3A, B). During a two-day observation period, the adults of *Diartiger zhejiangensis* (Fig. 3E, F) were totally ignored by the ant workers, and vice versa. This may have been a consequence of the disturbance of being transported and housed in the artificial environment.

#### Etymology.

The specific epithet refers to the province where the type locality of the new species lies.

### Modified couplets of the key (Yin and Li 2013) to *Diartiger* males from China

**Table:** 

2	Fourth antennomeres (Fig. 2A; Yin and Li 2013: 372, Fig. 2A) more than twice length of third antennomeres	2a
–	Fourth antennomeres (Yin and Li 2013: 372, Fig. 3A; Nomura 1997: 99, Fig. 4 ‘km.’) less than twice as long as third antennomeres	3
2a	Mesofemora with a sharp ventral spine at base; mesotibiae with a triangular spine at middle (Yin and Li 2013: 372, Fig. 2C), metacoxae (Yin and Li 2013: 372, Fig. 2D) with a small tubercle at ventral margin. (East China: Anhui)	*Diartiger dentatus* Yin & Li, 2013
–	Mesofemora and middle area of mesotibiae (Fig. 2C) lacking spine, metacoxae (Fig. 2D) lacking tubercle. (East China: Zhejiang)	*Diartiger zhejiangensis* sp. n.

## Supplementary Material

XML Treatment for Diartiger zhejiangensis
